# Spatial Distribution and Growth Patterns of a Common Bivalve Mollusk (*Macoma calcarea*) in Svalbard Fjords in Relation to Environmental Factors

**DOI:** 10.3390/ani14233352

**Published:** 2024-11-21

**Authors:** Alyona E. Noskovich, Alexander G. Dvoretsky

**Affiliations:** Murmansk Marine Biological Institute of the Russian Academy of Sciences (MMBI RAS), 183038 Murmansk, Russia

**Keywords:** *Macoma calcarea*, benthic ecology, environmental factors, growth patterns, spatial distribution, Barents Sea, Svalbard

## Abstract

Ongoing warming in the Arctic has led to significant sea-ice loss and alterations in primary production, affecting all components of the marine food web. The considerable spatial variability of near-bottom environments around the Svalbard Archipelago renders the local fjords promising sites for revealing responses of benthic organisms to different environmental conditions. We investigated spatial variations in abundance, biomass, and growth parameters of the common bivalve *Macoma calcarea* in waters off western Spitsbergen and identified two distinct groups of this species: one composed mainly of cold-water stations from Storfjorden (Group I) and the other comprising warmer-water stations from Grønfjorden and Coles Bay (Group II). Within these groups, the mean abundance, biomass, production, and mortality accounted for 0.2 and 429 ind. m^−2^, 20 and 179 g m^−2^, 18.5 and 314 g m^−2^ year^−1^, and 0.22 and 0.10 year^−1^ respectively. The size–frequency and age–frequency distributions were biased towards smaller and younger specimens in Group I, while Group II displayed more even distributions. The maximum ages were 11 and 21 years, respectively. The mollusks from cold water were significantly smaller than their same-aged counterparts from warmer water. Two groups of *Macoma* were identified: slow-growing individuals with a rate of 1.4 mm and fast-growing individuals with a growth rate of 1.8 mm. Most population parameters were higher than those observed in the Pechora, Kara, and Greenland Seas. Redundancy analysis indicated water temperature as the main driving factor of abundance and biomass, while the latter was also influenced by the presence of pebbles. Our findings provide new insights into the growth patterns and spatial distribution of *Macoma* at high latitudes and confirm that this species can serve as a reliable indicator of environmental conditions.

## Simple Summary

The observed warming trend in the Arctic has prompted investigations into the potential impacts on local communities, particularly in regard to the loss of sea ice and altered primary production. This study looked at *Macoma calcarea* bivalves, a common species found in near-bottom environments around Svalbard. The mollusks could be split into two groups. The first group was mostly made up of individuals from cold-water stations in Storfjorden. The second group was from warmer-water stations in Grønfjorden and Coles Bay. The two groups had different numbers, sizes, and growth patterns. In colder waters, the mollusks were smaller. Some grew faster than others. Most of the population parameters were different from those in the Pechora, Kara, and Greenland seas. Water temperature was the main reason for the differences in abundance and biomass. Pebbles also had an effect on biomass. Our findings show that *Macoma calcarea* can be used to monitor the environment in the Arctic.

## 1. Introduction

Despite the harsh environmental conditions in the Arctic, recent findings have indicated a significantly higher Arctic benthic diversity than initially assumed [1]. In fact, over 90% of the documented Arctic marine invertebrate species inhabit the sea floor as benthic species in contrast to the lower diversity of planktonic species [2] and fish [3]. The overall known benthic species richness amounts to over 4600 species [4,5]. The highest numbers of benthic species are found in areas where cold polar and temperate waters mix, such as the Barents or Bering Seas, and off the coast of West Greenland [6]. In terms of region, the greatest number of known species is found in the Barents Sea, partly due to the extensive research conducted in the past and partly due to its enrichment by boreo-Atlantic species [7]. The high productivity of the Barents Sea, resulting from the interaction of cold Arctic and warm Atlantic waters, supports the extensive fisheries of Atlantic cod, haddock, capelin, golden redfish, beaked redfish, Greenland halibut, red king crab, snow crab, and northern shrimp as well as considerable aquaculture potential in the coastal zone [8,9].

The Barents Sea is currently undergoing significant warming at a rate approximately three times the global average [10], with recent winter warming far surpassing that of summer despite pronounced seasonality. One distinguishing characteristic of the Barents Sea is the presence of sea ice, which exists permanently at high latitudes and seasonally at lower latitudes during winter [11]. The most significant indicator of warming is the dramatic loss of sea ice: Its summer extent has declined by nearly 50% during the past decade [12], and the Arctic Ocean has experienced a regime shift from multi-year ice to a predominately seasonal and thinner ice cover [13]. The decrease in sea-ice cover is further amplified by increased heat fluxes entering the Barents Sea through the Fram Strait [14]. The elevated ocean temperatures contribute to delaying the formation of sea ice in the fall [11]. Considering ecosystem functioning, these patterns suggest a shift towards an earlier spring transition between sea-ice-covered and ice-free conditions. Consequently, climate change in the Barents Sea is not only affecting physical structures such as sea ice but is also responsible for numerous ecological changes in ecosystem functioning [15]. These changes encompass modifications to food-web structure, stability, and efficiency, especially affecting the components at the base of the food web [1].

In general, seafloor communities rely on food resources supplied from the overlaying water column [16]. This food supply is crucial for the viability and growth of benthic organisms and constitutes the primary limiting factor for seafloor communities [1]. Generally, on the Barents Sea continental shelf, benthic communities receive abundant food input from the water column and consequently play a more significant role in system productivity and carbon cycling compared to communities at lower latitudes [1,17]. The two primary sources of production at high latitudes are sea-ice algae and phytoplankton [1,18]. The effects of prolonged warming and its consequences for primary production are expected to persistently influence the structure and function of marine benthos [19,20]. This, in turn, may further impact predators such as shrimp, crabs, fish, birds, and marine mammals [21].

Benthic organisms are deemed reliable indicators of environmental conditions due to their predominantly sedentary lifestyles and relatively long lifespans, which are conducive to detecting time-integrated responses to external forces on individual or community levels [22,23]. However, information about the responses of some abundant taxa to climate change remains limited. Bivalves are considered excellent indicators of environmental conditions due to their sedentary or sessile lifestyles, suspension or deposit feeding, and high abundance [24]. One such species, *Macoma calcarea*, is a widespread boreal-arctic circumpolar deposit feeder that inhabits muddy and gravelly sediments [25,26]. This species has free-swimming pelagic larvae and typically attains a maximum shell length of about 45 mm through a life span of up to 15–26 years [27,28,29,30,31]. *Macoma calcarea* is considered one of the most common bivalves in the Arctic seas’ infauna, with aggregations frequently exceeding 100 ind. m^−2^ [30,31,32,33], occasionally reaching up to 6000 ind. m^−2^ or approximately 4 kg m^−2^ [34]. Various top predators, including starfish, crabs, seabirds, and marine mammals, prey on this species [35,36].

Research conducted in the northern Bering Sea and southeastern Chukchi Sea has shown that the abundance and biomass of *Macoma calcarea* directly correlate with water temperature, chlorophyll-a inventories, and total organic carbon [35], thus substantiating the applicability of this species as an indicator of Arctic habitat conditions [37,38]. Although bivalve mollusks’ growth characteristics have demonstrated response to environmental variations, the growth patterns of *Macoma calcarea* have not yet been extensively studied in relation to the diverse conditions found in high-latitude areas of the Barents Sea. The same is relevant for abundance and biomass estimates. This is due to the fact that the majority of these areas are difficult to access.

Svalbard is an archipelago known for the predominance of two water masses, Atlantic water (AW) and Arctic water (ArW). The coastal shelves and fjord systems receive AW masses due to wind-driven cross-shelf exchange processes through the West Spitsbergen Current [39]. The interaction between AW and ArW occurs on the shelf, where the latter is transported northwards by the Spitsbergen Polar Current, resulting in the formation of the West Spitsbergen Polar Front along the density gradient between the two water masses [40]. Consequently, the western and eastern regions of Western Svalbard exhibit distinct thermohaline characteristics [41]. This area, therefore, is suitable for assessing the effects of differing environmental conditions on benthic animals.

Our study aims to compare populations of *Macoma calcarea* inhabiting Svalbard waters and determine whether the abundance, biomass, production, mortality, and growth parameters of this mollusk can be utilized as indicators of environmental conditions within the benthic realm at high latitudes in the Barents Sea.

## 2. Materials and Methods

### 2.1. Study Area

**Figure 1:**
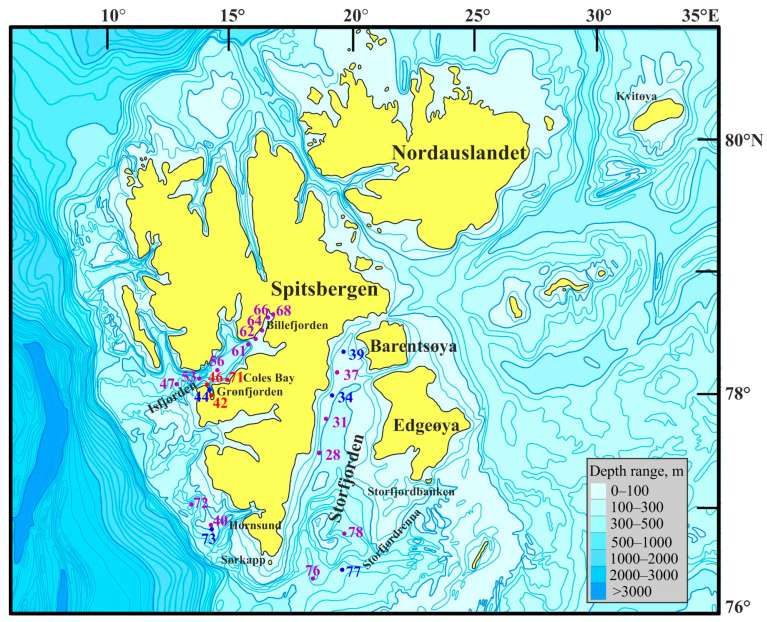
Location of sampling stations in Svalbard waters during the summer period of 2019. Violet—stations without *Macoma calcarea*; blue—Group I; red—Group II.

Most stations were sampled in the Isfjorden and Storfjorden systems (Figure 1).

Isfjorden, the largest fjord in western Spitsbergen, has a mean width of 24 km and extends approximately 100 km from its opening to the head of the side fjord, Billefjorden [41]. Encompassing a total area of 3084 km^2^ and a volume of 390 km^3^, its main basin (Isfjorden proper) is 70 km long and 200–300 m deep, oriented in a southwest–northeast direction, creating a 60° clockwise angle relative to the north direction. Grønfjorden, situated on the southwestern coast of West Svalbard, serves as the southwestern branch of Isfjorden. This meridionally elongated fjord covers more than 193 km^2^ in area [42]. Due to the absence of a sill, Grønfjorden exhibits continuous water exchange with Isfjorden due to tidal currents, resulting in significant variations in the local water mass structure on both seasonal and inter-annual timescales [43]. For example, in August 2016, intermediate water (IW) occupied depths between 40 and 130 m, while the underlying layer comprised AW. In the subsequent year, three distinct water layers were observed: IW at 25–80 m depths, transformed AW at 80–110 m, and AW below 120 m [43]. Long-term data from Ivanov and Svyashennikov [44] reveal a decreasing trend in ice exchange over the past decade, which mirrors the general patterns observed in the Barents Sea. The oceanography of this area is influenced by the comparatively warm and saline AW, which is transported northward along the shelf break by the West Spitsbergen Current. Coles Bay is a small bay located just northeast of Grønfjorden.

Hornsund, the southernmost fjord in Spitsbergen, is located on the west coast of the Svalbard Archipelago and occupies a total area of 311 km^2^. This area comprises the main basin (213 km^2^) and Brepollen (98 km^2^), the latter of which is situated in the inner part of the fjord, separated from the main basin by a submerged sill [45]. The entire drainage basin encompassed an area of 1200 km^2^, with 67% covered by glaciers. Cold ArW from the Barents Sea significantly influences Hornsund. Shelf water from the southwestern shelf of Svalbard forms strong density gradients as it gradually mixes with warmer AW, impeding the inflow of warm water into Hornsund compared to the northern Spitsbergen fjords that are predominantly influenced by warm AW [46]. However, Hornsund has experienced gradual warming in recent decades due to the increased influx of AW. Seasonally, multiyear ice covers Hornsund and the adjacent shelf area as it is transported around Sørkapp from the Barents Sea [46].

Storfjorden, a large semi-enclosed bay, is situated southeast in the Svalbard Archipelago between the islands of Spitsbergen, Barentsøya, and Edgeøya. It is delineated by a 120 m deep sill at approximately 77° N to the south, a shallow bank called Storfjordbanken in the southeast, and a submarine ridge in the southwest [47]. With a depth of nearly 190 m and a length of approximately 190 km, Storfjorden’s water mass exchanges below 70 m are guided by the seafloor morphology through a channel between 19° and 20°30′ E, extending from the sill to the trough of Storfjordrenna. The fjord predominantly receives cold and fresh ArW but is occasionally supplied with warm and saline AW, which enters the region following the cyclonic bathymetry due to Earth’s rotation. The local Polar Front, situated along the slope of Storfjordrenna [48], separates AW from ArW waters, resulting in rapid water mass transformations in Storfjorden with a renewal period of two months [49]. Numerous glaciers along the western coast contribute cold melt water and terrestrial sediments to the sea floor [47]. The predominance of winter-cooled water (WCW) in the near-bottom layer features low temperatures, high salinity, and minimal inter-annual variations in water temperature, occasionally reaching 1.8 °C in coastal regions.

### 2.2. Sample Collection and Analysis

Macrozoobenthos sampling was conducted at 23 stations (depth range 49–415 m) around western Svalbard (Figure 1) during the June–July 2019 research cruise aboard the R/V Dalnie Zelentsy. At each site, three replicate samples were collected using a Van Veen grab with a 0.1 m^2^ sampling area.

The acquired samples were washed through a 0.5 mm sieve and preserved in 4% neutral-buffered formalin, and sediment types were identified using standard methods [50]. Substrates were classified as follows: clay (particle size < 0.002 mm), silt (0.002–0.02 mm), sand (0.02–1 mm), gravel (1–10 mm), pebble (10–100 mm), stone (>100 mm), and shell (fragments of bivalve shells). Vertical profiles of water temperature and salinity were recorded using a CTD Sealogger SBE 19plus V2 (Sea-Bird Scientific, Bellevue, WA, USA).

In the laboratory, the benthic samples were washed once more and fixed in 75% ethanol solution before examining them under an MBS-10 stereomicroscope to identify specimens of *Macoma calcarea*. The mollusks (*n* = 191) were measured for shell length to the nearest 0.1 mm using a caliper or an ocular bar of the stereomicroscope. Each mollusk was then dissected and examined under a Mikmed-6 light microscope at 100× magnification. The mollusks were classified as males, females, or juveniles based on the presence or absence of gametes and their number and structure. Specifically, juvenile mollusks had no gametes, males had either mature spermatozoids or immature spermatocytes, and females had mature oocytes [51,52]. Abundance and biomass values were calculated for each station.

Production rates of *Macoma calcarea* were calculated using the formula proposed for stationary populations by Maksimovich and Pogrebov [53] as follows:

$$P=(\frac{N_{t}+N_{t-1}}{dx})(W_{t}-W_{t-1})$$

where *N_t_* and *N_t_*_−1_ represent the quantities of individuals at ages *t* and *t* − 1, while *W_t_* and *W_t_*_–1_ denote the mean individual weights of mollusks at ages *t* and *t* − 1, respectively. Additionally, a P/B ratio was determined, which is the production of mollusks at each age divided by their mean biomass.

Mortality rates (*M*, year^−1^) were assessed based on inter-annual changes in abundance across generations, using the formula provided by Maximovich and Guerasimova [54]:

$$M=\frac{\ln N_{1}-\ln N_{2}}{t_{2}-t_{1}}$$

where *N_t_* and *N_t_*_−1_ signify the quantities of individuals at time *t* and *t* − 1, respectively.

Age determination of bivalves based on external shell morphology is known to be challenging due to difficulties in determining the correct number of annual rings [27]. At the same time, there are a number of criteria that allow additional rings to be distinguished among annuals with greater certainty [55,56]. The possibility of using external shell morphology to determine the age of *Macoma calcarea* has been demonstrated for specimens from the White, Barents, and Kara Seas [29,30,57,58]. In this study, we used only those specimens for which the identification of growth rings was possible with a small uncertainty, i.e., in which the rings were rather clearly visible. To study the individual growth pattern, we measured shell length during annual growth delays and estimated an annual growth rate from the difference between adjacent final shell lengths in a consecutive series of ages [59]. We calculated group growth parameters (mean age series) by averaging individual growth characteristics, taking the shells of a few dead individuals into account in the age estimation process.

Given that the age series of *Macoma calcarea* do not indicate growth slowdown with age [60], we used a simple linear growth model as follows [58]:

$$L_{t}=a+bt$$

where *L_t_* is the shell length (mm) at time *t* (year), *a* is the slope, and *b* is the intercept.

### 2.3. Statistical Analysis

Principal component analysis (PCA) and one-way PERMANOVA based on the Euclidean distance matrix were used to determine the similarity of environmental conditions between stations with and without *Macoma calcarea*. To distinguish spatial variation in community structure, a cluster analysis was conducted based on group average linkage classification using the normalized Euclidean distance matrix of abundance, biomass, and size-frequency distribution data. The aforementioned metric permitted the processing of the variables with disparate units within a single complex. For this analysis, we used the stations where *Macoma calcarea* individuals were present. Similarities between station groups, as determined through hierarchical clustering, were examined using PERMANOVA. We also compared mean values between clusters using a one-way ANOVA after checking the data for normality and heterogeneity of variances using the Shapiro–Wilk and Levene’s tests, respectively. Differences in environmental variables between station groups were compared using Kruskal–Wallis test (KWT), as the data had non-normal distribution. Chi-square tests were conducted to examine differences in percentage data between station groups.

To reveal groups with different growth rates, we compared growth age series using the method proposed by Maximovich [60], which entailed pairwise comparison and clustering of growth age series through the analysis of residual variances with respect to growth models. We estimated the significance of variance distinctions using Fisher’s F-statistic (F), with the F/Fcr ratio (ratio of Fisher’s F-statistic to the critical F value at *p* < 0.05) serving as a measure of distance between compared age series. A value of F/Fcr < 1 indicated no significant differences between the compared age series. Clustering was conducted using the weighed pair–group average method. Since this analysis required a minimum of four measurements per age class, we selected 25 individuals with 4–5 clearly visible growth rings. In addition, we compared group growth data for ages 0–11 between station groups delineated by cluster analysis for spatial communities using an analysis of covariance (ANCOVA) after testing the data for assumptions required for parametric analyses.

To investigate the relationships between local environmental variables and the abundance and biomass data of *Macoma calcarea*, a redundancy analysis (RDA) was performed. This method was chosen based on preliminary detrended correspondence analysis, which revealed that the length of the first axis was <3 standard deviation units, indicating the linear ordination method as preferable to alternative techniques. The environmental dataset used in the analyses comprised temperature, salinity, depth, and substrate properties, while two distinct response variable datasets were employed. These datasets included abundances and biomass calculations for juveniles, mature females, and mature males of *Macoma calcarea*. Before including the selected environmental variables in the RDA, we tested our data for collinearity by calculating variance inflation factors (VIFs). As the analysis revealed VIFs of less than 5, all potential predictors were included in the final model. A Monte Carlo permutation test (n = 999) was carried out to reveal the explanatory variables that best explained the abundance and biomass data. All ordinations were conducted using CANOCO for Windows v. 4.5.

Other calculations were performed using NCSS PASS v. 2004 and PAST 4.12 software packages.

Mean values are presented with standard errors.

## 3. Results

**Figure 2:**
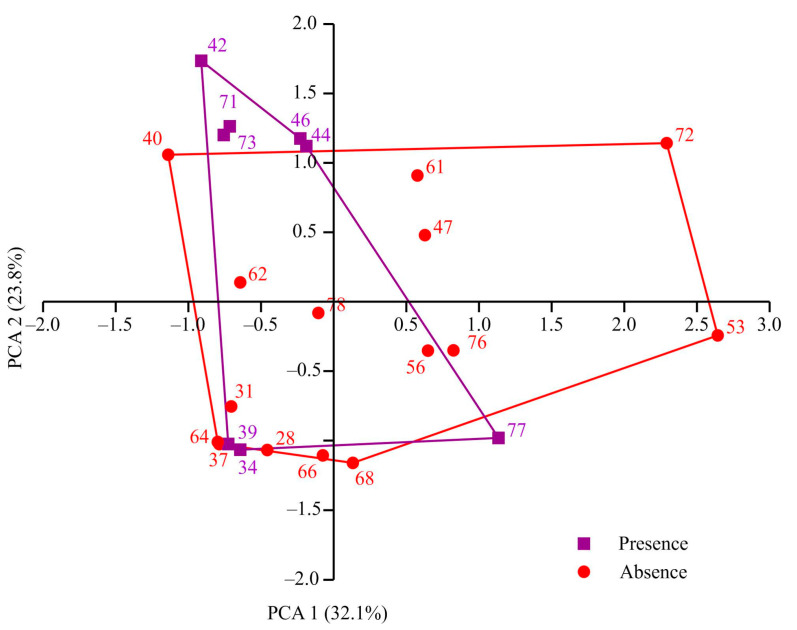
PCA plot showing distribution of sampling stations in relation to environmental variables in Svalbard fjords.

The sampling stations encompassed depths ranging from 49 to 419 m. Water temperatures varied between −1.88 and 3.4 °C, and salinity levels ranged from 34.6 to 35.35. Individuals of *Macoma calcarea* were found at eight stations, the majority of which were situated along the western coast of Spitsbergen. However, no mollusks were observed in Billefjorden and at the majority of deep-water stations. PCA separated sampling stations along axis 1, which explained 32.1% of the total variation, by depth (factor loading 99.9%), with deeper stations (most stations where *Macoma calcarea* was not recorded) on the right (mean depth 179 ± 28 m) and shallower stations on the left (123 ± 23 m). Axis 2 explained 23.8% of the total variation and separated stations by water temperature (factor loading 98%) so that most stations from warmer waters where *Macoma* was present were located in the upper part of the plot (Figure 2).

However, due to overlapping station distributions, PERMANOVA showed no significant differences between stations with and without *Macoma calcarea* (F = 1.83, *p* = 0.193).

Within the stations inhabited by *Macoma calcarea*, the abundance fluctuated from 6.6 ± 0.3 ind. m^−2^ (stations 44 and 73) to 306.6 ± 1.8 ind. m^−2^ (station 42), with an average of 79.5 ± 30.6 ind. m^−2^. Biomass also varied, ranging from 0.2 ± 0.02 g m^−2^ (station 73) to 429.2 ± 18.7 g m^−2^ (station 71), averaging a value of 100.5 ± 55.5 g m^−2^.

**Figure 3:**
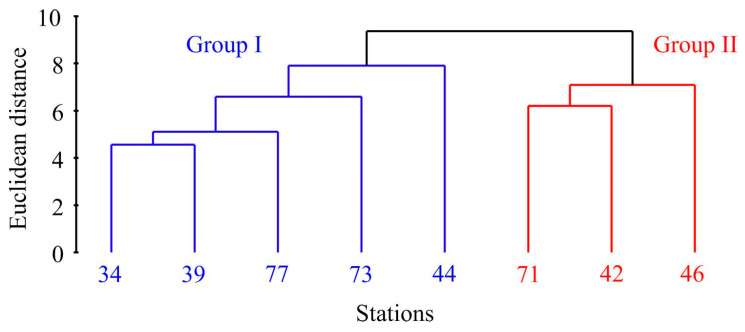
Dendrogram resulting from clustering performed on the normalized Euclidean distance generated from *Macoma calcarea* population data in Svalbard waters.

Cluster analysis of the abundance, biomass, and size data revealed two distinct groups of stations (Figure 3).

The first cluster, Group I, consisted of stations primarily located in the cold waters of Storfjorden (stations 34, 39, and 77) alongside two additional stations—one in front of Hornsund (station 73) and the other in Grønfjorden (station 44). Group II comprised stations 42 and 46 located in Grønfjorden and station 71 from Coles Bay. PERMANOVA confirmed the significance of this differentiation (F = 13.1, *p* = 0.018). More specifically, Group I stations displayed significantly lower abundance, biomass, shell length, and age values (Table 1).

**Figure 4:**
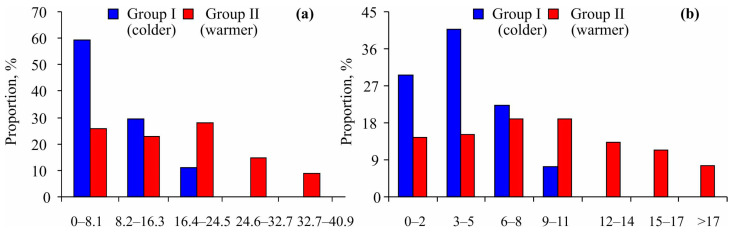
Size–frequency (**a**) and age–frequency (**b**) distributions of *Macoma calcarea* from different station groups delineated by cluster analysis in Svalbard waters.

Furthermore, the size–frequency distribution of *Macoma calcarea* showed a bias toward smaller specimens in Group I, while mollusks from Group II were distributed more randomly within their size classes (Figure 4a). A chi-square test indicated significant differences between the two groups (χ^2^ = 17.62, *p* = 0.001). Similarly, younger individuals were more prevalent at stations belonging to Group I (χ^2^ = 21.71, *p* = 0.001) (Figure 4b).

**Figure 5:**
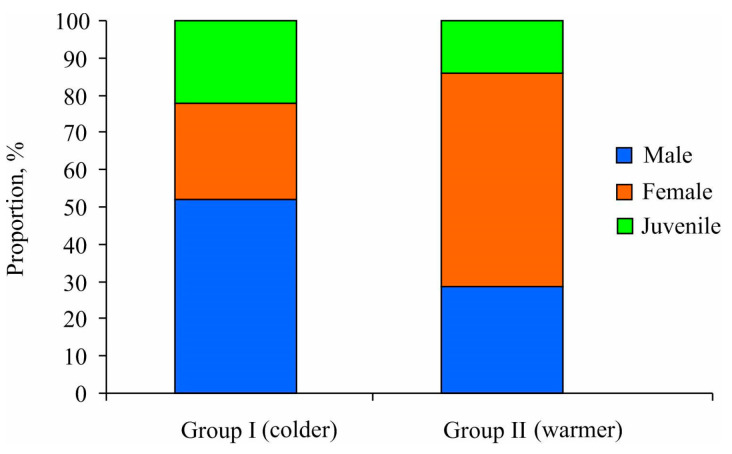
Proportional distributions of mature and immature individuals of *Macoma calcarea* from different station groups delineated by cluster analysis in Svalbard waters.

Additionally, the chi-square test results demonstrated that mature specimens predominantly constituted both station clusters, accounting for 77.8% and 87.1% in Groups I and II, respectively (*p* < 0.05) (Figure 5). Group I exhibited a sex ratio biased toward males (male/female = 2:1), albeit without significant differences due to the small sample size (χ^2^ = 2.38, *p* = 0.123). In contrast, Group II demonstrated a higher prevalence of females compared to males (male/female = 1:2) (χ^2^ = 15.56, *p* < 0.001). For males, the minimum size at maturity of *Macoma calcarea* males was determined to be 5.2 mm in shell length for Group I and 4.7 mm for Group II. Conversely, for females, this size was consistently 6 mm regardless of cluster group.

The annual production rates of *Macoma calcarea* were estimated to be 18.5 and 314 g m^−2^ at stations from Group I and II, respectively. Corresponding P/B ratios amounted to 0.96 and 1.15. Mortality rates for mollusks from Group I varied from 0.18 to 1.6 year^−1^, averaging 0.22 ± 0.18 year^−1^. Mortality was highest among one-year-old individuals, diminishing in subsequent years and increasing again at eight years of age. In Group II, fluctuations in mortality rate persisted as mollusks aged from zero to higher values, with the highest mortality rates observed in the 1-year, 3-year, and older-than-19-years age classes. The rates ranged from 0.10 to 1.79 year^−1^, with an average of 0.10 ± 0.22 year^−1^.

Concerning environmental variables, only water temperature exhibited significant differences between these two clusters. Specifically, water temperatures were lower at stations belonging to Group I (range: −1.79 to 2.65 °C; mean ± SE: 0.05 ± 1.03) compared to Group II (range: 2.75 to 3.4; mean ± SE: 2.97 ± 0.22) (Kruskal–Wallis Test, H = 5.00, *p* = 0.025). Simultaneously, Group I stations tended to possess greater depths (136 m vs. 99 m) and had a less frequent presence of pebbles among other substrate types (60% versus 100%).

**Figure 6:**
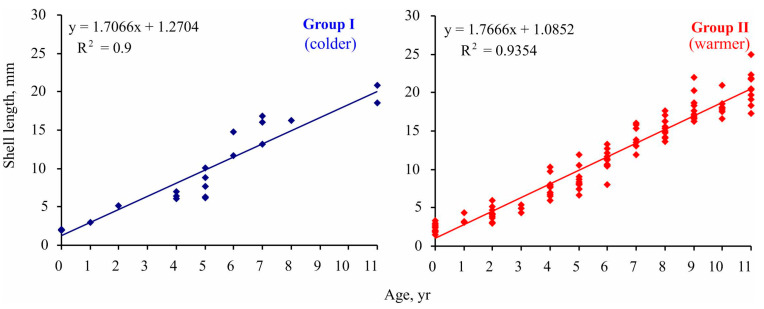
Size-at-age data for *Macoma calcarea* from different station groups delineated by cluster analysis in Svalbard waters.

The implementation of Maximovich’s method revealed significant differences in individual growth rates of *Macoma calcarea* in Svalbard fjords. Specifically, the size of the fifth growth ring ranged from 6 to 11 mm, and clustering delineated two groups: slow-growing individuals with a growth rate of 7 mm per five years and relatively fast-growing mollusks with a growth rate of 8.5 mm per five years. Although specimens belonging to these groups were found in both station complexes, the group growth pattern corresponded with the main findings for Clusters I and II. As depicted in Figure 6, in each age class, mollusks from Group II reached a larger size than specimens from Group I, a difference that was statistically confirmed (linear slope for group I = 1.706 ± 0.124 < linear slope for group II = 1.767 ± 0.045; ANCOVA, F = 1674.82, *p* < 0.001).

The maximum lifespan was 11 years in Group I and 21 years in Group II.

**Figure 7:**
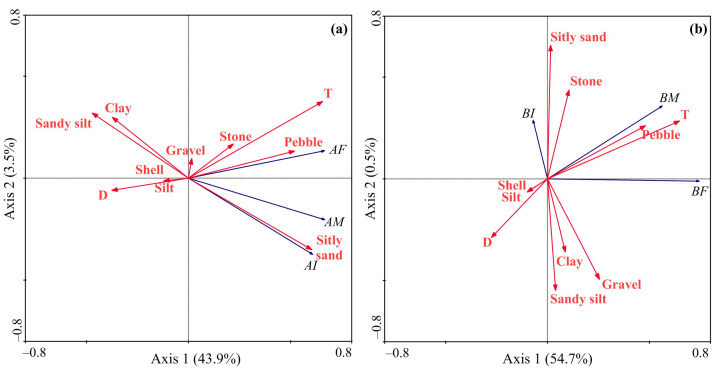
Ordination of samples by redundancy analysis with respect to *Macoma calcarea* abundance (**a**) and biomass (**b**) and their relationships with environmental variables in Svalbard fjords. The proportions of the total variability explained by the first two axes are given. Biological variables: AI—abundance of immature mollusks; AF—abundance of females; AM—abundance of males; BI—biomass of immature mollusks; BF—biomass of females; BM—biomass of males. Environmental variables: D—depth; T—temperature; S—salinity; Silt, Clay, Gravel, Pebble, Sandy silt, Stone, Silty sand, and Shell—substrate types.

The RDA model, based on the abundance data of *Macoma calcarea*, demonstrated that the first and second axes accounted for 47.4% of the total variance. Axis 1 exhibited strong positive correlations with temperature, silty sand, and pebble presence, separating stations with low or zero and high abundance, while Axis 2 had a negative association with silty sand presence and differentiated stations with female + juvenile dominance from male-dominated stations (Figure 7a). The RDA based on the biomass data generated a model where the first two axes elucidated 55.2% of the total variation (Figure 7b).

Almost all of this variation was explained by Axis 1, which differentiated sampling stations according to high (right side, adult mollusks) and low biomass values (left side, juvenile mollusks) and showed positive correlations with temperature and pebble presence and negative correlations with depth. The forward selection procedure revealed that water temperature was the only primary contributor to the observed variations in abundance data. Regarding biomass, the Monte-Carlo test identified water temperature and pebble presence as significant driving factors (Table 2).

## 4. Discussion

Svalbard waters exhibit considerable heterogeneity in terms of habitat conditions, including variations in temperature, salinity, sediments, and related properties such as primary production and carbon flux from upper water layers to the seafloor [41,47]. Near-bottom water layers are occupied by different types of water masses, resulting in favorable or less favorable conditions for bivalve mollusks. For instance, heat loss to the atmosphere, along with corresponding sea ice formation and brine release to the water below, are known to be driving processes of winter convection in Svalbard waters that promote the production of WCW [41]. During our study period, this water mass was observed in Billefjorden and Storfjorden, explaining the zero or very low abundance and biomass values of *Macoma calcarea* at these locations. Hornsund is significantly influenced by cold waters of Arctic origin, whose interactions with warmer waters foster the existence of intermediate water (IW). This water mass provides favorable temperature conditions for our bivalves, but the local habitats were sparsely colonized by *Macoma*, indicating that the thermal regime alone cannot explain this pattern. The western coast of Spitsbergen receives significant input of warm AW [41]. This type of water mass was registered at station 42, providing favorable conditions and the highest abundance of *Macoma calcarea* in this region. Transformed AW prevailed at station 46 in Grønfjorden and station 71 in Coles Bay, leading to high abundance and biomass values. Station 44 from Grønfjorden had surprisingly low biomass despite favorable water temperature, supporting the idea that other factors, including trophic conditions or biotic interactions [30], may be responsible for population trends of this bivalve mollusk. Indeed, the locations with the presence of pebbles, which are indicators of terrigenous organic matter input through intense currents, were found to be colonized by *Macoma* at high densities. It should also be noted that populations of sessile benthic organisms in Grønfjorden typically demonstrate a patchy distribution [42], which may further explain the reduced abundance and biomass levels at certain sites.

In our study area, we identified two groups of stations exhibiting clear differences in population characteristics of *Macoma calcarea*. Shallower stations with elevated water temperatures and suitable substrate and feeding conditions showed mollusks with higher abundance and biomass, larger shell length, and age (Table 1).

The abundance and biomass values of *Macoma* populations found around western Spitsbergen were similar to those in waters off Novaya Zemlya, where the abundance ranged from 27 to 197 ind. m^−2^, and biomass varied from 4 to 351 g m^−2^ [61]. Substantial spatial variations of these values were found in both regions, reflecting the complex local oceanography and circulation patterns. In contrast, populations of *Macoma calcarea* inhabiting the Pechora Sea demonstrated lower maximum (193 ind. m^−2^ and 57.5 g m^−2^) and average (40 ind. m^−2^ and 12 g m^−2^) abundance and biomass levels, with only weak variations in these indices. The presence of less dense populations in the Pechora Sea may reflect lower salinity values in that region and harsh ice conditions associated with a low impact of warmer and more productive AW on this area [62] compared to higher latitudes in Svalbard [56]. Indeed, the Pechora Sea is influenced by the cold coastal White Sea and Pechora currents, leading to low primary production values comparable to those in cold ArW [21]. Severe environmental conditions may also explain the lower biomass of *Macoma* (3–59 g m^−2^) in Disco Bay, Greenland [27] as compared to our data for the Svalbard areas affected by AW and TAW.

We found a predominance of smaller and younger specimens at sites belonging to Cluster I, where colder water temperatures resulted in slower growth rates (Figure 4). A similar result was registered for the White Sea [33]. The growth rate and lifespan of *Macoma calcarea* from Svalbard waters was also higher under favorable conditions (Cluster 2). Similar results have recently been published for Novaya Zemlya, where life spans were lower at reduced temperatures and higher at elevated temperatures [31]. The maximum lifespan of 21 years in Svalbard waters was comparable to 18–20 years registered for this species in the southwestern Kara Sea [57], shorter than 26 years near the Novaya Zemlya Archipelago [31], and longer than 14 years in the Baltic Sea [63], 15 years in some regions of the Kara and eastern Barents Seas [30,64], and 17 years in western Greenland [27]. At the same time, the growth rate of *Macoma calcarea* in the Kara Sea was less dependent on environmental conditions than in other locations [58].

Mortality is a pivotal attribute reflecting the population dynamics of a species and can be employed to compute the production rate of animals, thereby providing a clearer comprehension of their life history and habitat selection. Nevertheless, limited information is available concerning the mortality of bivalve mollusks in general, and only a few studies have reported mortality rates for *Macoma calcarea*. Our findings revealed that mortality of this species was two times lower at 2.75–3.4 °C (0.10 year^−1^) compared to stations where water temperature ranged from −1.79 to 2.46 °C (0.22 year^−1^). In contrast, in waters off Novaya Zemlya, annual mortality rates constituted 0.45 year^−1^ at 0.6–1.3 °C and 0.12 year^−1^, with temperatures ranging from −1.3 to −0.4 °C [61]. These observations indicate that factors other than water temperature influenced mortality in *Macoma calcarea* populations from the Barents Sea. In the White Sea, annual mortality varied from 0.37 yr^−1^ [29] to 0.58–0.84 year^−1^ [65]. Lisitsyna et al. [29] identified extensive variation in this parameter across size classes, ranging from 0.06 to 0.67 year^−1^ and described by a U-shaped curve representing mortality rates across size groups. Simultaneously, Gerasimova and Maksimovich [65] recorded an increase in the maximum mortality rate correlating with rising depth, from 1.06 year^−1^ at 8–10 m through 1.39 year^−1^ at 10–15 m to 1.75 year^−1^ at 40 m. In Disco Bay, mortality rates of *Macoma calcarea* demonstrated notable spatial variation, ranging from 0.2 to 0.58 year^−1^. The highest rates occurred at ages 1 and 5 years, while the lowest at ages 3–4 years [27]. It should be noted that mortality rates were calculated using a similar methodology.

Yearly growth rates of *Macoma* in our study were 1.4 and 1.8 mm for slow- and rapid-growing groups, respectively, averaging 1.5 mm. This value is similar to that registered in Disco Bay, Greenland [27], and the eastern Barents Sea [66] at higher than the 1 mm rate reported for the Kara Sea [30,57] but lower than 2.1 mm in waters off Novaya Zemlya [31]. Interestingly, in the Pechora Sea, at lower latitudes, 2-year-old mollusks had a shell length of 2 mm, i.e., half the size of those at stations belonging to Group II in Svalbard. Extremely slow growth rates in the first years of life, such as those observed in the Pechora and Kara Seas, have been proposed to reflect harsh temperature conditions associated with long ice-cover periods [30], and the higher growth rates that were evident for *Macoma* during our study at high latitudes may be attributable to the significant inflow of AW into the Isfjorden system, promoting prolonged ice-free periods. Higher abundances of larger mollusks have also been reported for the Bering Sea under optimal conditions [35].

We found a female-dominated population of *Macoma calcarea* at warmer stations in Svalbard as well as juvenile mollusks at high densities. This indicates intense reproductive processes in this population. For comparison, Petersen [27] found a balanced sex ratio in Disco Bay. Less favorable habitat conditions in the latter region also resulted in later maturation (the minimum size of a mature mollusk was 9 mm) [27] in comparison to our study (4.7–6 mm). The warming trend in the Arctic may have a beneficial impact on *Macoma* populations. Our study demonstrated higher growth rates and abundances of this species at warmer sites and under more favorable conditions. As a result of warming, this species may expand its range and occupy larger areas. An additional factor that can facilitate further development of this bivalve is a stronger influx of organic matter associated with longer ice-free periods at higher latitudes [67].

Thus, the large-scale spatial variations in abundance, biomass, and growth patterns of *Macoma calcarea* clearly reflect spatial variations in environmental conditions, among which the thermal regime seems to be most important. The latter assumption is supported by our RDA data for Svalbard fjords, according to which the water temperature explained 20 and 23% of the variation in abundance and biomass, respectively. The positive role of temperature in driving the abundance and biomass of this species was reported by Goethel et al. [35], who studied correlations between environmental variables and population characteristics of *Macoma calcarea* and registered a direct relationship between abundance and biomass and near-bottom temperature. In addition to temperature conditions, we found that the presence of pebbles was also positively correlated with biomass. High pebble content is usually associated with the input of terrigenous material and intense water circulation [68]. Both factors appear to be positive for *Macoma*. Terrigenous sediments are rich in carbon and may provide an additional food source for adult mollusks. A recent laboratory study conducted by Waga et al. [69] showed that the continuous input of fresh phytoplankton is more important for the growth of this mollusk than the total amount of phytoplankton reaching the seafloor. Thus, intense water circulation ensures the supply of fresh microalgal cells to *Macoma calcarea* at relatively shallow sites. It should also be noted that substrate properties can also directly affect the abundance and distribution of this species. In the Pechora and Bering Seas, *Macoma* have been found to prefer silty sand sediments and >5 phi grain size, respectively [30,35]. Our RDA ordination plot was in accordance with this pattern, showing a close association with silty sand and abundance and biomass of young mollusks (Figure 7b). Silt and clay showed a negative association with the abundance and biomass data, and this may explain the extremely low indices found at stations with optimal temperature conditions in Hornsund and Grønfjorden, where these types of substrates were predominant.

Our RDA models explained approximately 50% of the total variation in the abundance and biomass of *Macoma calcarea* in Svalbard waters, indicating that additional factors were involved in regulating the population dynamics of this bivalve mollusk. It is possible that currents may exert an influence on the population of *Macoma calcarea*. Field studies have demonstrated that the abundance of bivalves exhibits a significant correlation with physical variables linked to wind/wave activity and sediment resuspension [70]. Additionally, the transport of juvenile bivalves has been shown to fluctuate in response to tidal conditions [71]. Laboratory studies involving juvenile bivalves demonstrated that their dispersal rate frequently increases with rising velocity [72]. It can be postulated that the absence of *Macoma* at sites with optimal temperature conditions is attributable to an unfavorable current regime that impedes their dispersal and colonization of local habitats. Further research is required to elucidate the role of currents in shaping the *Macoma* populations in Svalbard waters. In addition, food quality and availability are of great consequence for benthic organisms [1,67]. While these parameters are directly dependent on temperature [73], additional organic matter sources may vary between locations, enhancing the productivity of habitats through terrestrial run-offs and melting water [74]. Furthermore, biotic interactions may also be responsible for fluctuations in the abundance and biomass of *Macoma calcarea*. Prior research has demonstrated that an overlap in the food spectra of different cohorts may result in intraspecific competition between adult and juvenile mollusks [75]. Competition for space has been extensively documented in the literature for various bivalve species [33]. It is also possible that interspecific competition may be a factor, but a recent study showed that in the Kara Sea, this factor had a negligible impact on local populations of *Macoma calcarea* [58]. Further studies are needed to test the contribution of other abiotic factors and biotic interactions to the population structure of *Macoma* in Svalbard fjords.

## 5. Conclusions

The fjords of western Spitsbergen, including Grønfjorden, Hornsund, and Storfjorden, are spatially separated and exhibit unique hydrographic characteristics, resulting in spatial variations in the abundance, biomass, and growth patterns of *Macoma calcarea*, associated mainly with local thermal regimes. The sites occupied by cooled water masses showed lower or zero biomass and abundance values of this mollusk, whereas at warmer waters, *Macoma* exhibited substantially higher levels of these parameters. Moreover, these levels were higher than at some lower-latitude locations affected by the inflow of colder water masses. Both individual and group growth rates were faster in Grønfjorden and Coles Bay as compared to Storfjorden, also exceeding the values reported for other regions under sub-optimal conditions. Water temperature was found to be the most important driving factor for abundance and biomass. In addition, sites with the presence of pebbles had higher biomass of *Macoma*, reflecting more favorable food supply at such locations, most likely due to the input of organic matter with terrigenous material. Our study showed *Macoma calcarea* population characteristics to be reliable indicators of environmental conditions at high latitudes. The data presented in this study are important for the understanding of marine ecosystem functioning and biogeochemical processes in high-latitude regions, as *Macoma calcarea* play an essential role in biogeochemical nutrient cycling, food webs, and ecosystem productivity, and changes in their populations may have significant implications for the broader ecosystem functions and services in the Arctic.

## Figures and Tables

**Table 1:** Population characteristics of *Macoma calcarea* in station groups defined by cluster analysis in Svalbard waters.

Parameter	Group I (Colder)				Group II (Warmer)				ANOVAS Tatistics	
	X	SE	Min	Max	X	SE	Min	Max		
									F	*p*
Abundance, ind. m^−2^	3.0	1.4	0.2	8.1	263.0	87.2	134.2	429.2	16.66	0.006
Biomass, g m^−2^	19.9	5.9	6.6	36.6	178.8	64.8	96.6	306.6	10.99	0.016
Shell length, mm	9.9	1.7	6.8	16.4	19.5	3.9	12.1	25.1	7.19	0.036
Mean age, yr	4.8	0.7	3	7	11.3	2.3	7	15	11.05	0.016

Note: Min—minimum; Max—maximum; X—mean; SE—standard error; F—Fisher’s ratio; *p*—probability level.

**Table 2:** List of environmental variables that contributed to the RDA models based on the abundance and biomass of *Macoma calcarea* in Svalbard fjords.

Variable	LambdaA	F	*p*	Variable	LambdaA	F	*p*
Abundance				Biomass			
T	20	5.09	0.020	T	23	6.30	0.012
Pebble	11	3.44	0.051	Pebble	13	3.88	0.045
Silty sand	5	1.43	0.221	Clay	9	3.06	0.105
Shell	7	2.19	0.156	Silty sand	3	1.21	0.287
D	2	0.67	0.407	D	5	1.81	0.181
Clay	2	0.50	0.501	Stone	1	0.31	0.608
Stone	0	0.11	0.829	Gravel	1	0.25	0.657
Gravel	0	0.02	0.967	Shell	0	0.09	0.765
Silt	1	0.04	0.871	Silt	0	0.06	0.775

Note: LambdaA—explained variation (%); F—pseudo F-ratio; *p*—probability level; D—depth; T—temperature; Silt, Clay, Gravel, Pebble, Sandy silt, Stone, Silty sand, and Shell—substrate types.

## Data Availability

The data presented in this study are available on request from the corresponding author (the data are not publicly available due to privacy restrictions).
